# Anti-Inflammatory and Antimicrobial Actions of Vitamin D in Combating TB/HIV

**DOI:** 10.1155/2014/903680

**Published:** 2014-07-02

**Authors:** Anna K. Coussens, Adrian R. Martineau, Robert J. Wilkinson

**Affiliations:** ^1^Clinical Infectious Diseases Research Initiative, University of Cape Town, Observatory, Western Cape 7925, South Africa; ^2^Blizard Institute, Barts and The London School of Medicine, Queen Mary University of London, London E1 2AB, UK; ^3^MRC National Institute for Medical Research, UK Medical Research Council, London NW7 1AA, UK; ^4^Department of Medicine, Imperial College London, London W2 1PG, UK

## Abstract

Tuberculosis (TB) disease activation is now believed to arise due to a lack of inflammatory homeostatic control at either end of the spectrum of inflammation: either due to immunosuppression (decreased antimicrobial activity) or due to immune activation (excess/aberrant inflammation). Vitamin D metabolites can increase antimicrobial activity in innate immune cells, which, in the context of HIV-1 coinfection, have insufficient T cell-mediated help to combat *Mycobacterium tuberculosis* (MTB) infection. Moreover, maintaining vitamin D sufficiency prior to MTB infection enhances the innate antimicrobial response to T cell-mediated interferon-*γ*. Conversely, vitamin D can act to inhibit expression and secretion of a broad range of inflammatory mediators and matrix degrading enzymes driving immunopathology during active TB and antiretroviral- (ARV-) mediated immune reconstitution inflammatory syndrome (IRIS). Adjunct vitamin D therapy during treatment of active TB may therefore reduce lung pathology and TB morbidity, accelerate resolution of cavitation and thereby decrease the chance of transmission, improve lung function following therapy, prevent relapse, and prevent IRIS in those initiating ARVs. Future clinical trials of vitamin D for TB prevention and treatment must be designed to detect the most appropriate primary endpoint, which in some cases should be anti-inflammatory and not antimicrobial.

## 1. Introduction

In recent years vitamin D has become a buzzword in disease prevention and treatment. There is an ever-expanding list of communicable and noncommunicable diseases being associated with vitamin D deficiency, including types 1 and 2 diabetes mellitus, rheumatoid arthritis, cardiovascular disease, osteoporosis, multiple sclerosis, depression, irritable bowel disease, asthma, colorectal, lung and breast cancers, upper respiratory tract infections, tuberculosis (TB), and HIV/AIDS progression and mortality [1–6]. No longer is vitamin D considered solely a regulator of calcium and bone homoeostasis; it is now recognized to have a diverse range of physiological functions, including cellular differentiation, proliferation, activation, and death [7]. One reason for its pleiotropic actions is the fact that vitamin D differs from most other vitamins in that its primary active metabolite, 1*α*,25-dihydroxyvitamin D (1,25[OH]_2_D), is a steroid hormone. Moreover, unlike many other vitamins which act as antioxidants or enzyme cofactors, vitamin D metabolites are ligands for the vitamin D receptor (VDR) and binding activates receptor-mediated signalling. Binding to the cell membrane receptor (mVDR) elicits rapid responses through activating intracellular signalling pathways, while binding to the nuclear receptor (nVDR) forms an activated ligand-dependent transcription factor complex that regulates the expression of more than 900 genes and microRNA [8–10].

Our research groups have been working on aspects of the genetic, molecular, and cell-mediated effects of vitamin D metabolites on* Mycobacterium tuberculosis* (MTB) infection* in vitro*,* ex vivo*, and* in vivo*, for the past 15 years. More recently, we have extended our research to investigate the interaction between vitamin D and TB in the context of HIV-1 coinfection and TB/HIV immune reconstitution inflammatory syndrome (IRIS). Here we review the impact of our findings in the context of the field and propose how we envisage vitamin D therapy that might have the greatest impact on reducing TB and HIV-1-associated incidence and morbidity.

## 2. Causes of Vitamin D Deficiency

### 2.1. Mechanisms of Vitamin D Acquisition

**Figure 1:**
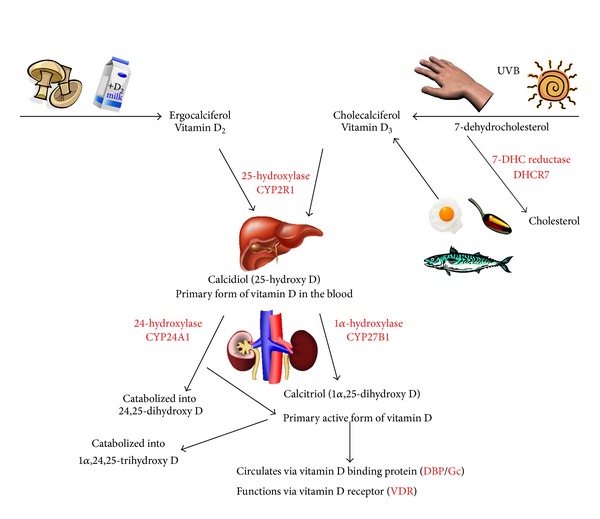
Vitamin D metabolism pathway. Proteins with genetic polymorphisms associated with vitamin D deficiency are highlighted (red).

Humans primarily obtain vitamin D through skin exposure to ultraviolet B (UVB) irradiation, cleaving 7-dehydrocholesterol, stored in the epidermis, and following isomerization forming vitamin D_3_. Alternate dietary sources of vitamin D_3_ (animal-derived) and vitamin D_2_ (plant- and fungus-derived) are also available, including oily fish, mushrooms, and supplemented dairy products; however their contribution to maintaining vitamin D sufficiency is much lower, except in the case of heavy supplementation or intake. The classic model of vitamin D metabolism involves its initial conversion in the liver to 25-hydroxyvitamin D (25[OH]D), the serum measure of vitamin D status, followed by 1*α*-hydroxylation in the kidney by the cytochrome P450 enzyme CYP27B1 to form 1,25[OH]_2_D, the active metabolite (Figure 1). 1,25[OH]_2_D acts on the kidney, the gastrointestinal tract, and the bones to regulate serum calcium concentration. It regulates its own catabolism via induction of the 24-hydroxylase CYP24A1 and its own metabolism through negative regulation of parathyroid hormone (PTH), which induces CYP27B1 in response to hypocalcaemia.

The increasing number of studies investigating diverse physiological functions of vitamin D has resulted in some contention in the field as how to define vitamin D deficiency; this is because the minimum activating concentration of vitamin D metabolites varies depending on the cellular process investigated [11]. Consensus has not been met, but the most frequently used definition of vitamin D deficiency (25[OH]D < 20 ng/mL or 50 nM) remains defined by the required serum concentration to maintain calcium homeostasis and to prevent secondary hyperparathyroidism [12].

### 2.2. Mechanisms of Vitamin D Deficiency

Decreased 25[OH]D levels can be caused by a variety of factors but primarily decreased exposure to UVB, which is particularly observed in geographical regions further from the equator, where UVB radiation is decreased due to the angle of the sun, which is exacerbated in winter or through behavioural avoidance patterns such as decreased time outdoors, increased sunscreen use, and full body covering for cultural reasons [13–15]. Non-UVB related causes include increased body mass index (BMI) due to sequestration of 25[OH]D in adipose tissue, decreased absorption of vitamin D in the intestines, increased 1*α*-hydroxylation of 25[OH]D, or increased catabolism of 25[OH]D and 1,25[OH]_2_D by CYP24A1 [16, 17]. These perturbations of vitamin D metabolism are influenced by genetics factors, including polymorphisms identified in CYP450 enzymes (Figure 1) [18] as well as disease states and medication [19–25].

#### 2.2.1. Infection Induced Vitamin D Deficiency

While vitamin D deficiency is associated with TB incidence and HIV-1 disease progression, vitamin D metabolism can also be modulated by MTB and HIV-1 infection. Toll-like receptor (TLR)1/2 stimulation by MTB antigens and HIV-1 gp120 peptides induces* CYP27B1* expression in infected macrophages* in vitro*. This accelerates 1,25[OH]_2_D production and subsequent* CYP24A1* expression. [26, 27]. Exacerbated by the fact that 1,25[OH]_2_D has a 4–8 hr half-life compared to the 15–18 days half-life of 25[OH]D [28, 29],* in vivo*, these infections may therefore result in exhaustion of available 25[OH]D if sufficient intake is not maintained.

#### 2.2.2. Drug Induced Vitamin D Deficiency

Drugs used to treat TB and HIV-1 also interfere with CYP450 enzyme levels. The first-line TB drugs rifampicin and isoniazid have been shown to decrease 25[OH]D in TB patients one month after therapy initiation and that this decrease is maintained after 6 months [19, 20, 30]. Protease inhibitors (PI) are also potent inhibitors of hepatic CYP450 enzymes [21]. Treatment of resting macrophages with HIV-1 antiretroviral PIs nelfinavir (NFV), indinavir (IDV), and ritonavir (RTV) also inhibits 25-hydroxyation and 1*α*-hydroxylation of vitamin D metabolites, consequently reducing bioactive 1,25(OH)_2_D [22]. While RTV has the greatest inhibition of CYP27B1, all PIs similarly inhibit CYP24A1 activity [22]. Conversely, while not having a direct effect on CYP450 enzymes, tenofovir (TFV), a nucleoside reverse transcriptase inhibitor (NRTI), is thought to mediate an indirect effect on vitamin D metabolism, through induced proximal renal tubular dysfunction, potentially affecting CYP27B1 activity and 1,25[OH]_2_D production. We have also shown that the TFV-associated increase in PTH levels is modified by sex and ethnicity [25]. Efavirenz (EFV) on the other hand, a nonnucleoside reverse transcriptase inhibitor (NNTRI), decreases serum 25[OH]D levels though inducing* CYP24A1* expression which effectively limits an increase in 25[OH]D following vitamin D supplementation [23, 24]. Together, these results suggest that TB and HIV-1 patients on chronic treatment are at risk of drug-induced vitamin D deficiency which may exacerbate preexisting infection-associated deficiency.

## 3. Vitamin D Deficiency, Genetic Variation, and TB/HIV Disease

**Figure 2:**
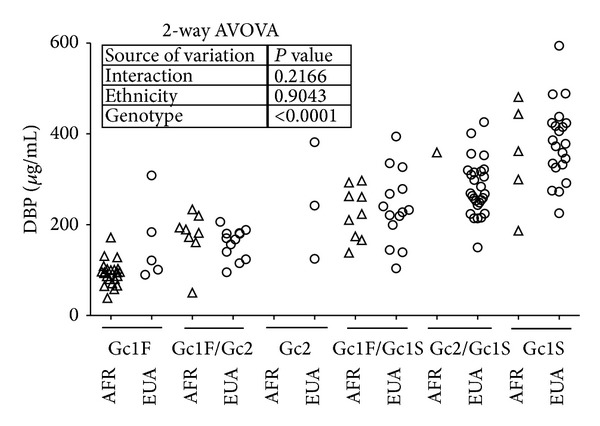
Serum vitamin D binding protein (DBP) concentration in patients with newly diagnosed pulmonary TB stratified by DBP Gc haplotype and ethnic group. Patients of Gc1F/1F haplotype had the lowest DBP concentrations and those with Gc1S/1S haplotype had the highest concentrations. Haplotypes frequency varied between ethnic groups of African ancestry (AFR) and Eurasian ancestry (EUA). The figure is adapted from Coussens et al. [33].

We first described the prevalence of vitamin D deficiency in TB patients in London, UK, in 2000 and demonstrated that individuals from certain populations who carry a VDR polymorphism at the Taq1 locus (rs731236), and are vitamin D deficient, have increased susceptibility to TB [31]. Further to this we identified a similar association with vitamin D deficiency and TB incidence in individuals carrying the vitamin D binding protein (DBP, the serum 25[OH]D transporter) Gc2 haplotype (T420K amino acid change) [32]. We subsequently demonstrated that, in TB patients from various ethnic backgrounds, DBP haplotypes are significantly associated with variation in serum DBP levels; patients of African ancestry who more commonly carry the DBP Gc1F haplotype have low circulating DBP levels, while Eurasians who commonly carry the Gc1S haplotype (D416E amino acid change) have high circulating DBP levels and individuals who are Gc2 or Gc1F-Gc1S have intermediate DBP levels (Figure 2) [33]. This observation is significant in light of the “free hormone hypothesis” which states that only DBP-unbound “free” 25[OH]D is bioactive; consequently serum DBP levels impact the level of bioactive 25[OH]D. In support of this hypothesis, recent studies have found that “free” 25[OH]D is more associated than total 25[OH]D to PTH concentration and bone density [34, 35]. Furthermore, different DBP haplotypes have different 25[OH]D binding affinities [36]. We assisted collaborators in demonstrating that monocytes treated with 25[OH]D and cultured in the presence of low-affinity Gc2 or Gc2-Gc1S DBP have a greater induction of vitamin D-associated gene transcription than those cultured with high affinity Gc1F DBP [37].

**Figure 3:**
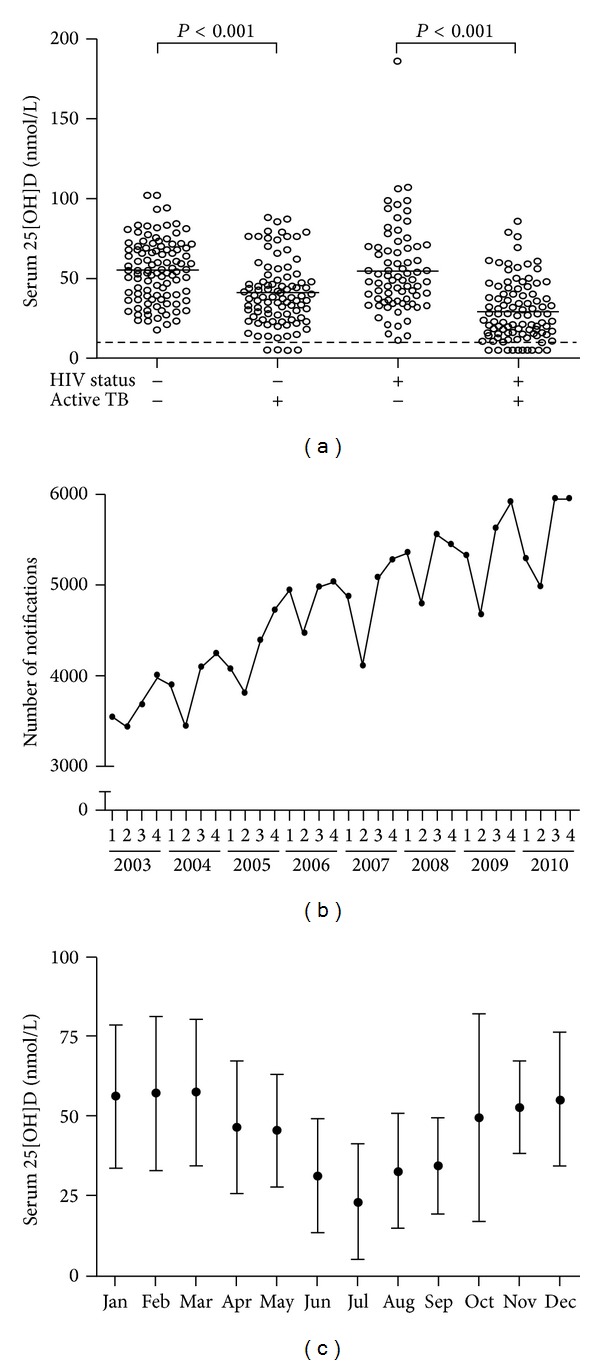
Active TB associates with vitamin D deficiency and seasonal fluctuations in Cape Town, South Africa. (a) Serum 25[OH]D concentration by HIV and TB status. Bars represent means. Dashed line represents limit of detection (10 nmol/L). (b) New TB notifications by quarter, 2003 to 2010. (c) Monthly variation in mean serum 25[OH]D concentration (all participants, *n* = 370). Error bars indicate SD. The figure is adapted from Martineau et al. [38].

Vitamin D deficiency has now been shown to be common in patients with active TB [31, 38] and is more prevalent in persons with latent TB who progress to active disease [39]. As mentioned, variation in UVB levels according to season and latitude impacts vitamin D levels [40]. Concordantly, we have shown that despite the general sunny climate of Cape Town, South Africa, at latitude 33°S, 75% of TB patients are vitamin D deficient, increasing to 86% in HIV-1 coinfected patients (Figure 3(a)) [38]. This is in contrast to Uganda, an equatorial country, where only 44% of hospitalized TB patients were found to be vitamin deficient, but again 83% of HIV-1 infected TB patients (with CD4 < 200 cells/mm^3^) were vitamin D deficient [41]. We also found that TB incidence in Cape Town oscillates seasonally, with the lowest rates occurring in autumn/winter, following peak 25[OH]D levels in summer (Figures 3(b) and 3(c)) [38]. Seasonality in TB notification has also been identified in Australia, USA, UK, and China with the strongest association in regions with larger seasonal fluctuation in UV index [42–45].

Apart from increased TB risk, vitamin D deficiency has also been identified to associate with increased risk of mortality and AIDS events in those with HIV-1, from a broad range of populations [46–48]. This association is further strengthened by the determination that two polymorphisms in the VDR (rs1544410 and rs2228570) associate with increased susceptibility to HIV-1 infection and rapid HIV-1 disease progression in adults and children [49–51]. An additional polymorphism in the 7-dehydrocholesterol reductase (DHCR7, rs12785878) also associates with rapid disease progression in children > 2 years [51].

## 4. The Inflammatory Balance in TB/HIV Progression

Upon initial infection with MTB, alveolar macrophages and dendritic cells (DC) recruit neutrophils, monocytes, T cells, and NK cells to activate cell-mediated killing. Upon recruitment, these cells encase the infected phagocytes, forming a granuloma that walls off the infected cells preventing MTB escape into surrounding tissue. A balanced inflammatory response from all these cells within these granulomas is vital to control excessive MTB replication and limit excessive inflammation, which is responsible for the immunopathology associated with TB morbidity. Loss of this finely balanced immune pressure, through various mechanisms of immunosuppression or immune activation, can therefore result in latent tuberculosis progressing to active disease [52, 53].

TB resurgence in sub-Saharan Africa is linked to the HIV-1 pandemic [54]. Coinfection with HIV-1 is thought to increase susceptibility to TB via a number of mechanisms, primarily through dysfunctional and decreased numbers of CD4^+^ T cells and impaired T cell activation by phagocytes [55–58]. Coinfection of peripheral blood mononuclear cells (PBMC) and macrophages with HIV-1 and MTB has been shown to mutually increase replication of both pathogens* in vitro* [59, 60]. MTB infection induces HIV-1 replication via a number of mechanisms, including upregulating host transcription factors nuclear factor-kappa B (NF-*κ*B) and nuclear factor of activated T cells-5 (NFAT5) which drive HIV-1 LTR transcription [60, 61]. Conversely, the effect of HIV-1 on the macrophage response is variable and subtle, modifying cytokine and chemokine production required for T cell recruitment and activation [59, 62].

Vitamin D metabolites have been shown to both induce cell-mediated antimicrobial activity against MTB and HIV-1 and have anti-inflammatory effects, regulating cytokine, chemokine, growth factor, and matrix metalloproteinase (MMP) expression [7]; consequently, the exact mechanism by which vitamin D may help to prevent and treat TB and HIV-1 remains unclear. More likely, its dual roles will have varying degrees of impact depending on the stage of infection and treatment.

## 5. Antimicrobial Effects of Vitamin D in relation to MTB and HIV-1 Infection

### 5.1. Alternate Vitamin D Receptor Activation

The first mechanisms identified by which 1,25[OH]_2_D_3_ mediated innate cellular control of MTB infection involved induction of nitric oxide, NAPDH-dependent oxidases, and phagolysosome fusion [63–65]. Vitamin D was shown to mediate its effect on the latter two functions via phosphoinositide 3-kinase (PI3K) signaling, suggesting that the antimicrobial effects of vitamin D were mediated by the mVDR [63, 64]. However, through the utilisation of specific mVDR and nVDR inhibitors we determined that the predominant mechanism of vitamin D anti-MTB activity was actually mediated via the nVDR and that this was associated with 1,25[OH]_2_D_3_ induction of cathelicidin antimicrobial peptide (CAMP) gene expression [66], the product of which is enzymatically cleaved to produce the active antimicrobial agent, cathelicidin (LL-37) [67].

### 5.2. Cathelicidin-Mediated Antimicrobial Activity

Vitamin D regulation of CAMP expression by MTB-infected monocytes was first demonstrated a year earlier, by Liu et al. [27] who also showed that LL-37 directly inhibits MTB viability in liquid culture. This gene regulation by vitamin D was explained by CAMP having three vitamin D response elements (VDRE) in its promoter [68]. In the same paper Liu et al. demonstrated that TLR1/2 stimulation of MTB-infected monocytes upregulates expression of VDR and CYP27B1 and that monocytes grown in the presence of 25[OH]D-replete serum upregulated CAMP expression. Thus, monocytes have the capacity to utilise serum 25[OH]D to induce intracellular antimicrobial gene expression [27].

Since its first identification in monocytic phagocytes, further studies have since reported that neutrophils, T cells, B cell, NK cells, DC, mast cells, and epithelial cells in the upper and lower respiratory tract also express CAMP [69–71]. This suggests that vitamin D metabolites could induce antimicrobial activity in a diverse range of cells in response to MTB and HIV-1 infection.

The bactericidal activity of cathelicidin is mediated by its ability to bind and disrupt bacterial cell wall phosphatidylglycerol monolayers [72]. However, recent work has shown that pro-LL-37 (hCAP18) also induces autophagy though upregulating expression of beclin-1 (BECN1) and autophagy protein 5 (ATG5), which mediate activation of p38 mitogen activated protein kinase (MAPK) and extracellular signal-regulated kinase (ERK) 1/2 signaling [73]. Autophagy has recently been identified as a key intracellular process to antagonize MTB-mediated inhibition of phagosome maturation, a key mechanism by which MTB subverts the innate immune response [74]. Autophagy also increases the production of bacterial degradation products in antigen presenting cells for pattern recognition receptor activation and presentation to the adaptive immune system [75]. Moreover, 1,25[OH]_2_D_3_-induced autophagy in monocyte-derived macrophages (MDM) has also been shown to restrict HIV-1 replication [76]. We have obtained similar results on HIV-1 replication using MDM differentiated in the presence of physiological concentrations of 25[OH]D_3_, although we hypothesize an alternate mechanism of action mediated by 25[OH]D_3_ regulating secretion of CCL chemokines which regulate HIV-1 replication (*manuscript in prep.*).

In general, all papers that report an effect of CAMP induction on colony forming unit (CFU) restriction demonstrate only a small effect, in the range of 2-fold [27, 66, 77, 78]. Moreover, almost all studies present the induction of CAMP expression with the insinuation that LL-37 is produced, thus mediating the restriction of growth. However proteinase 3 (PR3) which is required for LL-37 cleavage from the propeptide is predominantly expressed in neutrophils [67]. Therefore in single cell culture systems involving monocytic phagocytes, the likelihood of LL-37 production is low. Moreover, the only way of detecting LL-37 production is via Western blot which is rarely incorporated as a proof of concept and thus while authors interpret their results to reflect LL-37 production, in fact they may only be observing the effects of hCAP18.

### 5.3. Interferon-*γ* Regulation of the Vitamin D Response

**Figure 4:**
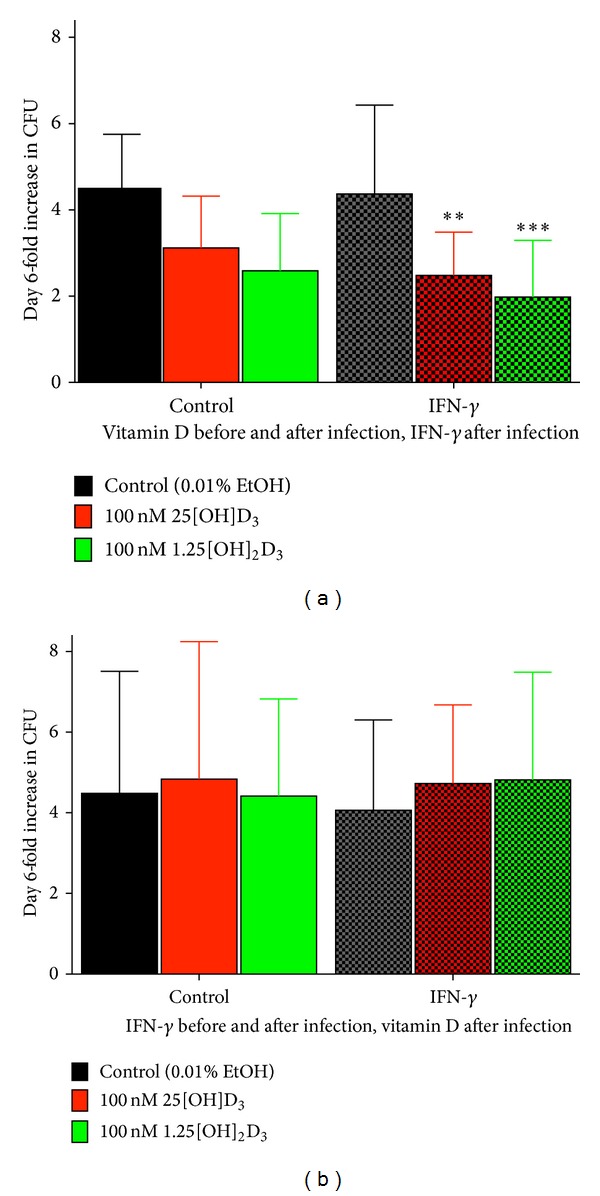
Interferon-gamma (IFN-*γ*) enhances the antimicrobial action of monocyte-derived macrophages (MDM) infected with* Mycobacterium tuberculosis* only in vitamin D sufficient conditions. (a) MDM differentiated in the presence of vitamin D significantly inhibits MTB growth in response to postinfection IFN-*γ* (200 IU/mL) treatment. This scenario represents the logical physiological process of infection response under vitamin D sufficient conditions. (b) However, pretreatment of MDM with IFN-*γ* (200 IU/mL) for 48 hr before infection and during infection in vitamin D deficient conditions does not enhance the response to vitamin D treatment after infection. Mean ± SD, *n* = three donors, each in triplicate.

The antimicrobial effects of vitamin D metabolites have also been shown to be increased by cotreatment with interferon-gamma (IFN-*γ*) as it can enhance macrophage levels of 1,25(OH)_2_D_3_ by antagonizing the ability of 1,25(OH)_2_D_3_ to induce CYP24A1 and suppress CYP27B1 [79, 80]. Work by Fabri et al. [78] showed that stimulating human monocytes with IFN-*γ* for 24 hours in 10% vitamin D-sufficient human serum increases the 1,25(OH)_2_D/24,25[OH]_2_D ratio, induces CAMP expression, and increases autophagolysosomal fusion but not when using vitamin D-deficient human serum. This is surprising given the fact that the final concentration of 25[OH]D in culture was only 10% of the serum concentration. However, we have similar results demonstrating that, in macrophages grown in the presence of 100 nM 25[OH]D_3_ during differentiation and throughout MTB infection (replicating physiological vitamin D sufficiency), IFN-*γ* treatment after infection enhances the response to vitamin D (Figure 4(a)). Conversely, IFN-*γ* treatment of MDM for 48 hr preinfection and during infection (in vitamin D deficient conditions) had no effect on vitamin D-mediated MTB growth restriction when 100 nM 25[OH]D_3_ was added after infection (Figure 4(b)). Together with the observations by Fabri et al. [78], these results suggest that maintaining vitamin D sufficiency prior to infection will enhance T cell-mediated innate cell responses during MTB infection.

### 5.4. Neutrophil-Derived Antimicrobial Peptides

In addition to CAMP induction, 1,25[OH]_2_D has also been shown to increase expression of another antimicrobial peptide beta-defensin 2 (DEFB4) [78, 81, 82]. In comparison to CAMP, the DEFB4 promoter only has one VDRE but two NF-*κ*B binding sites. Consequently, its expression is regulated by dual signaling mechanisms, with vitamin D a smaller component [81]. Moreover, while the effect of vitamin D metabolites on antimicrobial peptide expression is commonly studied in monocytes or macrophages* in vitro*,* in vivo* these peptides are more abundant in neutrophils and epithelial cells [83, 84]. We have demonstrated that, in whole blood (WB) assays, the greatest control on MTB growth is mediated by neutrophils and that the antimicrobial peptides human neutrophil peptide 1–3 (HNP-13) and neutrophil gelatinase-associated lipocalin (NGAL) (the latter in the presence of iron) elicit more efficient killing of MTB than LL-37 [85]. Therefore, to truly understand the* in vivo* related effects of vitamin D antimicrobial action, studies should focus on integrating* in vivo* studies and* ex vivo* analysis of mixed-cell cultures to better understand the antimicrobial actions of vitamin D.

### 5.5. *Ex Vivo* and* In Vivo* Antimicrobial Effects of Vitamin D

To investigate physiologically significant effects of vitamin D on MTB restriction in humans, we initially conducted a proof of concept trial, investigating the effect of a single bolus dose of vitamin D_2_ (100,000 IU), given to healthy TB contacts, on restriction of* Mycobacterium bovis* BCG-lux luminescence (which correlates with CFU [66]), in an* ex vivo* WB culture, 6 weeks after administration [86]. This assay ensured the presence of neutrophil-derived products, particularly PR3 for LL-37 production. This dose of vitamin D_2_ significantly increased participants 25[OH]D levels 6 weeks after administration and resulted in a significant restriction in BCG-lux luminescence 24 hr and 96 hr after infection of WB compared to blood taken before supplementation [86].

Based on these results we conducted a trial of high-dose adjunctive vitamin D_3_ (100,000 IU, fortnightly for 8 weeks) during intensive phase treatment of pulmonary TB patients, from diverse ethnic backgrounds, in London, UK [87]. There was a trend to reduction in median time to sputum culture conversion with vitamin D_3_ (36.0 days) compared to placebo (43.5 days, *P* = 0.14). In subset analysis, we identified a significant interaction between response to vitamin D_3_ and the VDR TaqI polymorphism, such that those homozygous recessive had a significant reduction in time to culture conversion with vitamin D_3_ supplementation. Furthermore, refining our analysis to the per-protocol subset, who amongst other criteria received >3 doses of vitamin D_3_ and was not HIV-1 infected or taking corticosteroids, vitamin D_3_ significantly reduced time to sputum smear conversion [88]. One reason for the significant response in the per-protocol cohort is likely greater and more frequent supplementation. We found that fortnightly doses of 100,000 IU (approx. 7,000 IU/day) only increased mean 25[OH]D, in the entire cohort, above the optimal threshold of 75 nM after 4 weeks. We hypothesize that a greater antimicrobial effect of vitamin D_3_ may be achieved with a higher, more frequent dose or through supplementation with 25[OH]D_3_ rather than vitamin D_3_ to rapidly raise serum 25[OH]D. Further trials are required to comprehensively conclude whether these alternative regimes will improve the efficacy of adjunct vitamin D_3_ to reduce time to culture conversion. However, despite the inconclusive effect of vitamin D_3_ on MTB clearance in our trial, we did find a significantly greater and widespread reduction in inflammatory markers in those receiving vitamin D_3_ during anti-TB therapy, compared to placebo, irrespective of VDR polymorphism [88]. This suggests that vitamin D's greatest role may be resolution of pathologic inflammation during TB treatment.

## 6. The Anti-Inflammatory Action of Vitamin D in relation to TB

The immunomodulatory effects of vitamin D have been extensively studied, even before the identification of the cell-mediated antimicrobial mechanism of vitamin D. The molecular link between vitamin D and the immune system was first identified in the early 1980s with the discovery that monocytes and macrophages express VDR, they can synthesize 1,25(OH)_2_D_3_ and that 1,25(OH)_2_D_3_ induces differentiation of monocytes into macrophage-like cells which have increased phagocytic, lysozyme, and migration activity [89–91].

### 6.1. Anti-Inflammatory Effects of Vitamin D

With regard to the anti-inflammatory roles of vitamin D during MTB infection, we first demonstrated that 1,25[OH]_2_D_3_ inhibits IFN-*γ*, TNF, and IL-12p40 expression and secretion from MTB-infected PBMC, while concomitantly inducing NOS2A and CAMP expression [66]. During treatment of pulmonary TB, we have demonstrated that vitamin D_3_ significantly inhibited secretion of proinflammatory cytokines IL-1RA, IL-6, IL-12p40, and TNF from WB stimulated with MTB peptides, ESAT-6 and CFP-10, while attenuating the reduction in IL-4, CCL5, and IFN-*α* observed during intensive-phase therapy [88]. Moreover, we found that vitamin D_3_ supplementation increased the lymphocyte : monocyte ratio, previously shown to be a marker of healing lesions [92], and accelerated the TB therapy-induced reduction in acute phase markers, erythrocyte sedimentation rate (ESR) and C-reactive protein (CRP), and IFN-*γ* and IFN-*γ*-simulated chemokines CXCL9 and CXCL10. While this may be useful during treatment, based on these observations, maintaining vitamin D sufficiency prior to infection could cause a decrease in proinflammatory responses during initial infection which is counterintuitive to a protective response. However, in those with latent TB or acute infection, vitamin D may modulate the inflammatory response to limit excessive inflammation associated with disease activation, while simultaneously enhancing antimycobacterial activity.

The exact mechanisms of vitamin D's anti-inflammatory effects are not yet fully delineated. It cannot be ruled out that the anti-inflammatory effects we observed in our vitamin D_3_ trial are due to enhanced bacillary killing. However,* in vitro* evidence suggests that vitamin D metabolites will have a direct effect of inflammatory responses* in vivo* as it has been shown that they directly inhibit MAPK and NF-*κ*B signaling. The expression of MAPK phosphatase-1 (MKP-1, which dephosphorylates activated MAPK) is upregulated in human monocytes by 1,25[OH]_2_D_3_ and this associates with increased binding of the nVDR and increased histone H4 acetylation at the VDRE in the MKP-1 promoter [93]. NF-*κ*B activity is similarly inhibited by 1,25[OH]_2_D_3_ which increases I*κ*B*α* levels and decreases its phosphorylation (the first step in degradation of the NF-*κ*B inhibitor) leading to decreased NF-*κ*B nuclear translocation and activity [94, 95].

We have also shown that 1,25[OH]_2_D_3_ induces IL-10 from PBMC* in vitro*, and vitamin D supplementation increases IL-4 secretion in WB MTB antigen stimulation assays, suggesting that vitamin D polarizes towards a Th2 response. Others have also shown that vitamin D metabolites induce regulatory T cell (Treg) differentiation and FoxP3 expression where seasonal vitamin D deficiency is associated with decreased Foxp3 expression by Tregs and that serum 25[OH]D levels correlate with Treg function [96, 97]. While it is unclear whether vitamin D regulates Treg function in TB, we have recent data showing that 25[OH]D_3_ induces expression of the natural innate inhibitor IL-37 in MTB-infected macrophages (*manuscript in prep.*). IL-37, a member of the IL-1 family (aka IL-1F7), has recently been identified to suppress macrophage TLR-induced cytokine and chemokine secretion, by between 92–98% for IL-1*α*, TNF, and IL-6 as well as IL-1*β*, IL-12, G-CSF, and GM-CSF to a lesser extent, without regulating IL-10 or IL-1RA. IL-37 primarily mediates its effect by translocating to the nucleus and forming a complex with Smad3 as well as reducing phosphorylation of p38 MAPK and STAT1–4. Moreover, secreted IL-37 is able to bind to the IL-18Ra chain, preventing IL-18Ra from recruiting the IL-18Rb chain and also bind to IL-18BP, antagonizing IL-18 mediated-responses [98, 99]. IL-37 may therefore be the fundamental mediator by which vitamin D elicits its broad anti-inflammatory effects.

### 6.2. Anti-MMP Effect of Vitamin D

Further to an effect of vitamin D on inflammatory cytokine secretion, we have also demonstrated that 1,25[OH]_2_D_3_ inhibits expression, secretion, and/or activity of MMP which are linked to cavitation and granuloma formation in TB due to their ability to degrade all components of the extracellular matrix (ECM) [100–103]. In MTB-infected monocytes and PBMC 1,25[OH]_2_D_3_ treatment inhibits MMP-1, MMP-7, and MMP-10, while it constitutively inhibits MMP-9, irrespective of infection [104]. Under the same conditions, we observed increased IL-10 and prostaglandin E2 (PGE2) secretion, both known to negatively regulate MMP secretion/activity [105, 106]. The effect of 1,25[OH]_2_D_3_ on MMP-7 and MMP-9 has also been seen using PBMC from TB patients [107] and we have found that adjunctive vitamin D_3_ during anti-TB therapy suppresses plasma MMP-9 levels [88]. Vascular remodeling is also a crucial component of lung destruction in active TB [108]. Human vascular endothelial cells also metabolize 25[OH]D_3_, while treatment with 1,25[OH]_2_D_3_ decreases endothelial cell proliferation and increases cellular adhesion to endothelial cells [109]. Therefore, adjunctive vitamin D may also help to resolve and prevent lung tissue destruction during infection as well as resolve pathology and restore lung function during TB treatment.

While the majority of work on MMP focuses on tissue destruction, MMP also plays a role in regulating inflammation. As endopeptidases they have been shown to release TNF and IL-6 from cell surfaces and inactivate IL-1*β*. The release and degradation of TNF and IL-1 isoforms are potential feedback regulatory loops as both cytokines are implicated in MMP induction [110–112]. Moreover, MMP regulates cell recruitment, by processing of chemokines and through cleaving fragments of ECM which act as chemotactic signals for inflammatory cells, including elastin degradation products in lung parenchyma [113–117].

## 7. The Interaction between Vitamin D, HIV-1, and TB

### 7.1. Vitamin D Regulation of HIV-1 Transcription

The regulation of cytokines and chemokines by vitamin D not only impacts TB inflammation, but also has the potential to impact HIV-1 replication. While we have discussed the potential autophagy-mediated antimicrobial action of vitamin D on HIV-1 replication, the inhibition of TNF, IL-6, and CCL2 secretion by vitamin D and its metabolites* in vivo* and* in vitro* [66, 88, 93, 118] has the potential to reduce HIV-1 replication, through inhibiting NF-*κ*B-mediated HIV-1 transcription [60]. We are currently investigating whether vitamin D supplementation inhibits* ex vivo* HIV-1 replication in PBMC, via these mechanisms. Recently, 1,25(OH)_2_D_3_ was also found to regulate microRNA expression, including inducing miRNA-22, which targets NFAT5 [10]. As this transcription factor has been shown to regulate HIV-1 LTR transcription in macrophages, miRNA-22 induction may be an additional mechanism by which vitamin D inhibits HIV-1 replication [61]. While ARV is successful in decreasing HIV viral load and improving CD4 cell count, these observations suggest that vitamin D supplementation may have the ability to target clearance of the tissue viral reservoirs that ARV is unable to eradicate, potentially further decreasing TB risk in HIV-1 infected individuals.

In support of this, a recent prospective study of TB incidence in HIV-1 infected individuals initiating ARV therapy and receiving nonvitamin D containing multivitamins identified a significant correlation between vitamin D deficiency and incidence of pulmonary TB but not malaria or pneumonia [119]. Analysis of the same cohort also showed that vitamin D deficiency was significantly associated with increased all-cause mortality, compared to vitamin D sufficiency [120].

### 7.2. Vitamin D and TB/HIV Immune Reconstitution Inflammatory Syndrome

A frequent complication of ARV initiation in HIV-1 infected individuals with other opportunistic infections, including MTB and* Cryptococcus*, is the development of immune reconstitution inflammatory syndrome (IRIS). Paradoxical TB-IRIS occurs in TB patients who are clinically improving on anti-TB therapy, who begin ARV and develop paradoxical deterioration [121]. We have demonstrated that, in cross-sectional analysis, patients who develop TB-IRIS have a higher frequency of IFN*γ*-secreting T cells recognizing MTB antigens, compared to similar patients treated for both HIV-1 and TB who did not develop IRIS. However, over 8 weeks longitudinal analysis during ARV initiation, all patients, irrespective of IRIS development, have dynamic changes in the frequency of antigen-specific IFN*γ*-secreting T cells, suggesting that these changes are not causal [122]. We have since identified a myeloid-derived hyperinflammatory profile involving proinflammatory cytokines IFN-*γ*, IL-1*β*, IL-2, IL-6, IL-8, IL-10, IL-12p40, GM-CSF, and TNF and increased MMP-1,-3,-7, and -10 [123, 124]. This suggests that IRIS may develop due to overstimulation by MTB antigens of innate immune cells which could be inhibited by vitamin D.

In a study of the use of corticosteroid (CTC) therapy to prevent TB-IRIS, we found that while CTC use decreased CXCL10, IFN*γ*, IL-6, IL-8, IL-10, IL-12p40, IL-18, and TNF prior to ART initiation, it did not prevent IRIS onset, although it did decrease severity, and that severe vitamin D deficiency (total 25(OH)D < 25 nmol/L) was associated with higher baseline IL-6, IL-8, and TNF, irrespective of IRIS status [125]. IRIS development is also associated with higher MTB antigen load and lower CD4 cell count [121]. Therefore as vitamin D can have a positive impact on reducing bacterial and viral load, increasing Treg function and inhibit a broad spectrum of proinflammatory cytokines and MMP production it may be a more effective therapy than CTC to prevent IRIS and less subject to adverse events.

## 8. Future Potential of Vitamin D Therapy in the Context of TB/HIV

Vitamin D, having dual antimicrobial and anti-inflammatory properties, has the potential to be utilised for both prevention and treatment of TB to reduce TB morbidity and, in the context of HIV-1, decrease TB risk, IRIS incidence/severity, and HIV/AIDS disease progression. Therefore, what are the barriers to translate scientific experience to clinical practice? Primarily, it is a number of null trials of vitamin D which have been recently conducted [87, 126–128]. However, the findings from these unsuccessful trials remain ambiguous as in many cases an argument could be given that the dose, regime, or metabolite used was incorrect, that the baseline context of vitamin D deficiency was not investigated or reported (we know that vitamin D has the greatest effect in those most deficient [23]), that the populations were not geographically at risk of deficiency, or that due to the interaction of vitamin D with genetic polymorphisms which are population specific, supplementation may have differential benefits for various populations, which again are affected by location and diet.

However, the greatest reason for the lack of significant effect of vitamin D trials for TB may be that their primary outcome is a measure of the antimicrobial effect of vitamin D in combination with highly effective antimicrobial therapy. We propose that while vitamin D does have anti-MTB effects, in combination with highly effective anti-TB therapy, the effect is likely to be modest. By using larger more frequent bolus doses in larger trials conducted with sufficient powering, the effect will be detected. However, it is unlikely to be of sufficient magnitude to justify trials for treatment shortening in drug-sensitive disease. Rather, the most significant outcome from our trial of vitamin D_3_ during anti-TB therapy was the pleiotropic and expansive acceleration of resolution of inflammation and an increase in cellular ratios indicative of healing of lung pathology. We therefore suggest that future trials of adjunct vitamin D during TB treatment incorporate a primary endpoint relating to lung function and lung pathology, in addition to culture conversion.

Secondly, while the antimicrobial actions of vitamin D do not outweigh those of intensive-phase therapy, vitamin D does have the potential to act as preventative therapy in both TB contacts, who are not immunosuppressed but live in high burden settings, or in HIV-1 infected individuals with CD4 counts above the threshold for ARV initiation, and in those who are drug-resistant TB contacts for which isoniazid preventive therapy (IPT) is not efficacious.

Finally, the anti-inflammatory, combined with antimicrobial, effects of vitamin D may have dual roles in preventing and minimizing the severity of TB-IRIS. Giving vitamin D prior to ARV initiation may help to both further reduce the bacterial burden, as antigen load is the greatest known risk factor for IRIS development, and decrease the inflammatory environment. This could potentially limit both innate and adaptive immune cell activation during immune reconstitution which is the cause of morbidity.

Vitamin D is a potential cheap and widely available therapy which potentially has wide-ranging benefits for human health. We are only hindered in our ability to translate these benefits to the clinic by insufficient clinical trials evidence of efficacy. However, we will continue to trial vitamin D in diverse circumstances in the hope of potentially seeing successful outcomes, which will finally lead to clinical implementation.
